# Paradoxical Interaction between Ocular Activity, Perception, and Decision Confidence at the Threshold of Vision

**DOI:** 10.1371/journal.pone.0125278

**Published:** 2015-05-08

**Authors:** Aaron Schurger, Min-Soo Kim, Jonathan D. Cohen

**Affiliations:** 1 Department of Psychology, Princeton University, Princeton, New Jersey, United States of America; 2 Princeton Neuroscience Institute, Princeton University, Princeton, New Jersey, United States of America; 3 INSERM U992 / NeuroSpin, CEA-Saclay, Gif-sur-Yvette cedex, France; State University of New York Downstate Medical Center, UNITED STATES

## Abstract

In humans and some other species perceptual decision-making is complemented by the ability to make confidence judgements about the certainty of sensory evidence. While both forms of decision process have been studied empirically, the precise relationship between them remains poorly understood. We performed an experiment that combined a perceptual decision-making task (identifying the category of a faint visual stimulus) with a confidence-judgement task (wagering on the accuracy of each perceptual decision). The visual stimulation paradigm required steady fixation, so we used eye-tracking to control for stray eye movements. Our data analyses revealed an unexpected and counterintuitive interaction between the steadiness of fixation (prior to and during stimulation), perceptual decision making, and post-decision wagering: greater variability in gaze direction during fixation was associated with significantly increased visual-perceptual sensitivity, but significantly decreased reliability of confidence judgements. The latter effect could not be explained by a simple change in overall confidence (i.e. a criterion artifact), but rather was tied to a change in the degree to which high wagers predicted correct decisions (i.e. the sensitivity of the confidence judgement). We found no evidence of a differential change in pupil diameter that could account for the effect and thus our results are consistent with fixational eye movements being the relevant covariate. However, we note that small changes in pupil diameter can sometimes cause artefactual fluctuations in measured gaze direction and this possibility could not be fully ruled out. *In either case*, our results suggest that perceptual decisions and confidence judgements can be processed independently and point toward a new avenue of research into the relationship between them.

## Introduction

In the domain of perceptual decision making, a distinction can be drawn between two types of visually-informed decisions: “first-order,” such as the identification of a visual object or feature, and “second-order”, such as the estimation of the quality of the sensory evidence and/or the certainty of the first-order decision [1]. Neuroscientists have made considerable progress in trying to identify and characterize the neural phenomena that correspond to first-order [2] and second-order [3–5] decision processes, but the precise relationship between the two remains an open question.

A second-order decision is typically an expression of the degree of confidence in a decision, or confidence in the quality of the evidence on which a decision is based. In addition to humans [6], some non-human species, including apes [7], monkeys [3, 8], dolphins [8], and even rats [4] appear to be capable of behaviorally expressing their level of certainty (or uncertainty) about their decisions (for review see [9]). While there remains some debate as to whether these faculties qualify as “metacognition” [10], it is clear that accuracy and confidence are dissociable [5, 11–14].

Authors of prior work have adopted the convention of referring to first-order and second-order decisions as “type-1” and “type-2” decisions, respectively [1, 5, 15], and we will follow that same convention here.

In the study of type-2 decision making, it is important to distinguish between the overall degree of confidence, and the reliability of the confidence judgements. One’s degree of confidence can vary independently of one’s accuracy: one could, in theory, offer low confidence judgements more often than high ones and yet perform well on a task, or vice versa. In addition, the reliability of one’s confidence judgements, called type-2 sensitivity [1, 15], is also theoretically distinct from both accuracy and confidence, although there are some a-priori constraints on the relationship between type-1 and type-2 sensitivity [15]. Type-2 sensitivity refers to the degree to which the subject’s confidence judgements accurately estimate his probability of being correct, and this is what we are concerned with here. While type-1 and type-2 sensitivity can vary between individuals [5], the precise relationship between these two types of decision remains unknown. In particular there is an open debate as to whether these two types of decision rely on distinct neural processing pathways [16] or can be accounted for by different criteria applied to a single source of neural information [14, 17].

By using ocular activity as a covariate, we provide evidence that type-1 and type-2 sensitivity can be driven in opposite directions, suggesting that they may be subserved by distinct mechanisms. We continuously recorded gaze direction and pupil diameter during a cued fixation interval, while subjects performed a perceptual (type-1) decision-making task and also engaged in post-decision wagering. Post-decision wagering is a form of type-2 decision-making that is motivated by the prospect of winning money [18]. On each trial, after making a perceptual decision, the participant places a wager. The amount wagered is won or lost depending on the accuracy of the perceptual decision. Presumably, participants tend to wager more money when they are more confident in their decision. Accordingly, the amount wagered (a type-2 decision) provides a quantitative index of the confidence they had in their perceptual (type-1) decision. While post-decision wagering may be subject to biases such as loss aversion [19], one can rule out criterion effects by either testing for a shift in the proportion of high wagers, or by using unbiased measures of type-2 decisions based on signal-detection theory (SDT) [1, 15].

Using a two-alternative forced-guess [20] object-categorization task together with post-decision wagering, we found that the sensitivity of high wagers to correct decisions fell to zero (as a function of color contrast) before accuracy on the perceptual task fell to chance. Data from infrared eye tracking revealed that this dissociation was linked to a differential interaction between the steadiness of visual fixation (as measured by our equipment; see Discussion) and type-1 and type-2 judgements: greater variability in measured gaze direction [21] during stimulus presentation, while subjects were fixating, was associated with significantly more-accurate guessing, but significantly less-advantageous wagering.

Recall that “less advantageous wagering” does not necessarily imply “lower confidenÎ” (a simple shift in bias), but rather refers to the degree to which confidence matches accuracy, i.e. the ability to decide whether or not one’s own uncertain decision is likely to be correct. While type-2 sensitivity can be estimated using metrics based on signal detection theory [1, 15], these are aggregate measures and are thus not defined at the single-trial level, whereas the proportion of advantageous wagers (PAW) is. PAW will vary to some degree purely as a function of overall confidence, but for a constant or near-constant proportion of high wagers, advantageous wagering estimates the sensitivity of the type-2 judgement. We make use of PAW when a single-trial metric is called for, being careful to rule out effects due purely to changes in overall confidence.

## Materials and Methods

### Subjects

Subjects were Princeton University students who either signed up to perform the experiment for pay, or for course credit. A total of 14 subjects participated in the experiment (4 males, mean age 20 yrs). The first six (6) subjects performed the experiment before we had access to the eye-tracking equipment. An additional eight (8) subjects performed the experiment with eye tracking. The experimental protocol was approved by the Princeton University Institutional Review Board, and all subjects gave written informed consent before participating in the experiment. Data from three subjects were excluded from the behavioral data analyses (see below), and the eye-tracker data file was corrupt for one subject. Thus N = 7 for subjects contributing both behavioral and eye-tracker data, and N = 11 for subjects contributing behavioral data.

### Stimuli, procedures, and task

Stimuli were simple line drawings of faces and houses, 175 by 175 pixels, line width 9 pixels blurred with a Gaussian filter (radius 2 pixels) and rendered in exactly two colors using error-diffusion dither (Photoshop CS2, Version 9.0, Adobe Systems, Inc). There were a total of 24 stimuli, 12 faces and 12 houses. Stimuli were presented on a 15” LCD display (30.5 x 23cm, 1024 x 768 pixels, 60Hz refresh) at a viewing distance of 45cm, and subtending approximately 6° x 6° of viewing angle. Stimuli were presented dichoptically with the aid of a cardboard divider and prism lenses [22] (see S1 Fig). Two black square borders just large enough to frame the stimuli were displayed on either side of the vertical meridian. These were left on the screen at all times during blocks of trials, and stimuli always appeared within the frames.

**Fig 1:**
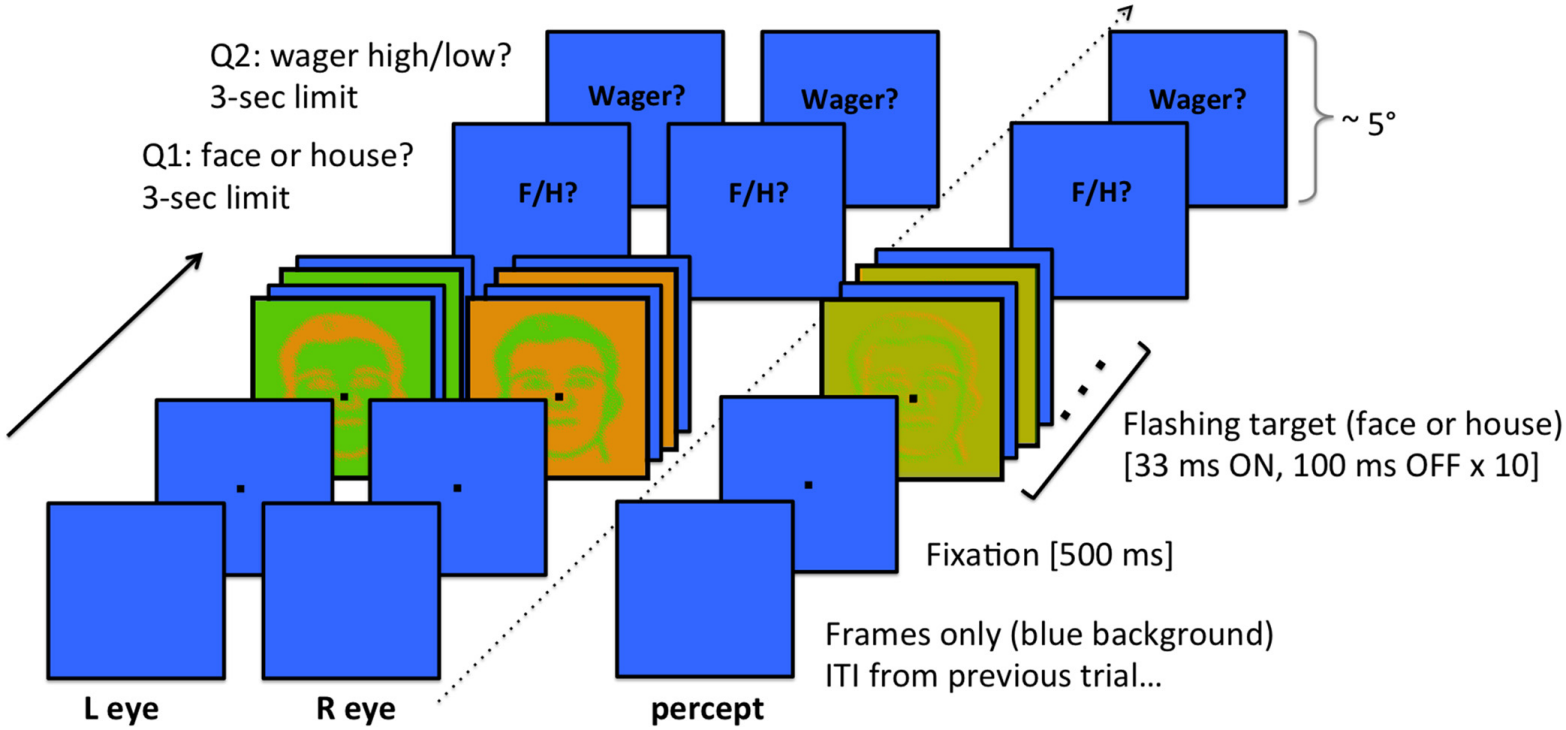
Trial sequence. Stimuli were presented dichoptically using prism lenses and a cardboard divider (see S1 Fig). To the left of the dotted line are the trial sequences from the point of view of each eye, and to the right of the dotted line is the sequence from the subjective point of view showing the resulting fused percept. The visibility of the object depended on the color contrast, which varied from trial to trial. 500ms before stimulus onset, a fixation point appeared (2x2 pixels; < 0.1°) cueing the subject to keep his/her gaze as steady as possible for the next two seconds. In order to limit the possibility of binocular rivalry [24, 25] the stimulus was flashed at a rate of 8 Hz (2 refresh cycles ‘on’ and 6 cycles ‘off’ at 60 Hz refresh rate), for a duration of 1.33 seconds. After stimulus cutoff, the fixation point disappeared and a text prompt appeared cueing the subject to respond (subjects were instructed to wait for the prompt before responding). The first question on each trial was to identify the category of the object by pressing either the ‘F’ (face) or ‘H’ (house) key on a computer keyboard with the left hand, guessing if necessary. Then a second prompt appeared cueing the subject to place a wager (‘high’ = 20¢ or ‘low’ = 5¢) on the accuracy of their immediately preceding perceptual decision, by pressing either the ‘1’ (low bet) or ‘2’ (high bet) key on the numeric key pad with the right hand. If the subject waited longer than 3 seconds to respond, the fixation point would disappear, the inter-trial interval would elapse, and then the next trial would begin. Subjects rarely took longer than 1.5 sec to respond.

We manipulated the visibility of the stimuli by reversing the figure-ground color assignment between the two eyes (dichoptic color masking or DCM [23–25]; Fig 1 and S1 Fig) and parametrically varying the color contrast. The two colors used in the line drawings were isoluminant mixtures of red and green at different levels of color contrast, yielding opposing shades of pale orange and pale yellow-green. A calibration was performed on the display device using heterochromatic flicker photometry [26] so that isoluminance could be maintained across a range of different levels of color contrast. Heterochromatic flicker photometry involves displaying a colored patch on the screen that alternates rapidly (e.g. at 15 Hz) between the two colors. The luminance of one color is adjusted up and down by hand in order to find the point at which subjective flicker is minimized. Because the apparent flicker is driven mainly by differences in luminance, then the point of minimum apparent flicker approximates the point of subjective isoluminance. RGB values (0 < = RGB < = 1) for the set of color pairs used on our display device ranged from [0.4 0.2745 0] to [0.4 3.647 0] for the shades of orange, and [0.2343 0.3414 0] to [0.3528 0.3869 0] for the shades of yellow-green (see S1 Table for the full set of RGB values used, but bear in mind that these were calibrated on our display device, and are included for illustrative purposes only—these are unlikely to be isoluminant on your display device). The screen background was blue and approximately isoluminant with the stimuli so that the onset of the stimulus would be less likely to provoke blinks or saccades. The lights were turned off in the testing room during the experiment.

Before the experiment, subjects practiced maintaining steady fixation by trying to sustain the disappearance of a blurred peripheral annulus (Troxler’s illusion [27]) for a few seconds at a time, repeatedly for two or three minutes. Subjects were instructed to maintain steady fixation during each trial, from the onset of the fixation point (black, 2 x 2 pixels, appearing 500ms pre-stimulus) through the disappearance of the stimulus (about 2 seconds in total). Once seated in front of the computer display (with cardboard divider and prism lenses) a dual-image random-dot stereogram was used to confirm dichoptic fusion [22].

The sequence of events during each trial (explained to subjects verbally; see Fig 1) was as follows: 500ms before stimulus onset, a fixation point would appear cueing the subject to keep his/her gaze as steady as possible for the next two seconds. In order to limit the possibility of binocular rivalry [24, 25] the stimulus was flashed at a rate of 8 Hz (2 refresh cycles ‘on’ and 6 cycles ‘off’ at 60 Hz refresh rate), for a duration of 1.33 seconds. After stimulus cutoff, the fixation point disappeared and a text prompt appeared cueing the subject to respond (subjects were instructed to wait for the stimulus to stop flashing before responding). On each trial the subject was to decide whether the object on the screen had been a face or a house, guessing if necessary, by pressing either the ‘F’ (face) or ‘H’ (house) key on a computer keyboard with the left hand. After each perceptual decision the subject placed a wager (‘high’ = 20¢ or ‘low’ = 5¢) on the accuracy of his/her own guess by pressing either the ‘1’ (low bet) or ‘2’ (high bet) key on the numeric key pad with the right hand. Overall winnings could not go below zero or above $20. The subject had a maximum of 3 seconds for each response (from the time of stimulus onset), but subjects rarely used more than half of that time. If the subject waited longer than 3 seconds to respond, the fixation point would disappear, the inter-trial interval would elapse (1.5–2.0 sec, varying randomly), and then the next trial would begin.

The experiment was run in blocks of 24 trials, with each block representing one cycle, in random order, through all 24 stimuli (12 face / 12 house). Before beginning the experiment, subjects performed three practice blocks with above-threshold “same color” stimuli, at three progressively lower levels of color contrast. After the practice blocks there were four experimental blocks with “opposite color” stimuli at one of four different levels of color contrast (chosen based on prior pilot testing) varying randomly from trial to trial.

### Eye tracking

Eye tracking was performed on 8 of the 11 subjects who participated in the experiment using an infrared eye-tracking system (Applied Science Laboratories, Bedford, MA), sampling at 60Hz. This particular eye-tracking device can resolve differences in relative eye position on the order of 0.25°. The eye-tracker data file for one subject was corrupted and could not be read, leaving N = 7 for eye-tracking analyses. During eye tracking, the subject’s head was secured using a chin/forehead rest (Applied Science Laboratories). Prior to each experiment, the eye tracker was calibrated on the 6° x 6° frame in which the stimuli would appear on the computer display. The eye-tracking camera was positioned just to the left of the cardboard divider, and monitored the subject’s left eye.

### Data analysis

#### Data inclusion criteria

Data were excluded from the analysis if the subject failed to score at least 90% correct on the 2^nd^ and 3^rd^ practice blocks, or there were too few high wagers to accurately estimate wagering performance. Three subjects (among the first six) were excluded from the behavioral data analyses for one or more of these reasons, leaving N = 11 for behavioral analyses.

#### Wagering

One way to analyze wagering behavior is to use signal-detection theory (SDT): a correct decision is treated as “target present” and a high wager placed on a correct decision is treated as a “hit”. ***d’*** can then be calculated as *zinv*(HR)—*zinv*(FAR), where *zinv* is the inverse normal distribution, HR is the hit rate, and FAR is the false-alarm rate (the 0.5 convention was applied whenever either HR or FAR was zero [28]). Intuitively this measure (type-2 ***d’*** or ***Wd’***, for “wagering d-prime”) attempts to estimate how well the subject made use of the high wagers that s/he placed, independently of the subject’s willingness to place high wagers [29]. In order to estimate ***Wd’*** with reasonable accuracy it is necessary to have at least a moderate number of high wagers, and subjects may avoid high wagers under uncertainty due to the phenomenon of loss aversion [19]. In an attempt to counter loss aversion, we told subjects “it is OK to bet *high* all of the time, but please avoid betting *low* all of the time—try to ‘go for it’ (i.e. bet *high*), even if you have only a vague hunch. It might help you to win more money.”

According to SDT, type-2 ***d’*** (***Wd’***) is expected to vary positive linearly purely as a function of type-1 ***d’*** (sensitivity on the first-order perceptual decision making task), and so any apparent differences in wagering sensitivity could simply be due to changes in the accuracy of the type-1 decisions. In order to control for this relationship Maniscalco and Lau [15] recently developed a metric for estimating the sensitivity of type-2 decisions, called “meta d-prime”, that is independent of the type-1 decision accuracy. We computed both measures in our analyses and they both gave qualitatively similar results (see below).

Wagering behavior can also be analyzed by labeling each wager as subjectively advantageous or disadvantageous: high wagers on correct decisions and low wagers on incorrect decisions are labeled as “advantageous”, and the proportion of advantageous wagers (*PAW*) can be computed [18]. *PAW* must be treated with caution since, unlike ***Wd’***, it is subject to decision biases such as loss aversion [19, 29]. However, it has the advantage of being applicable to single trials (***d’*** is strictly an aggregate measure). We make use of the “advantageous wager” metric (with due caution) when a single-trial measure is called for. Although both ***Wd’*** and *PAW* are problematic (and have been criticized [1, 19, 29]), the primary finding reported here does not depend on the particular value(s) that these measures take on, but rather on the way that they interact with ocular activity.

#### Eye tracker data

**Fig 2:**
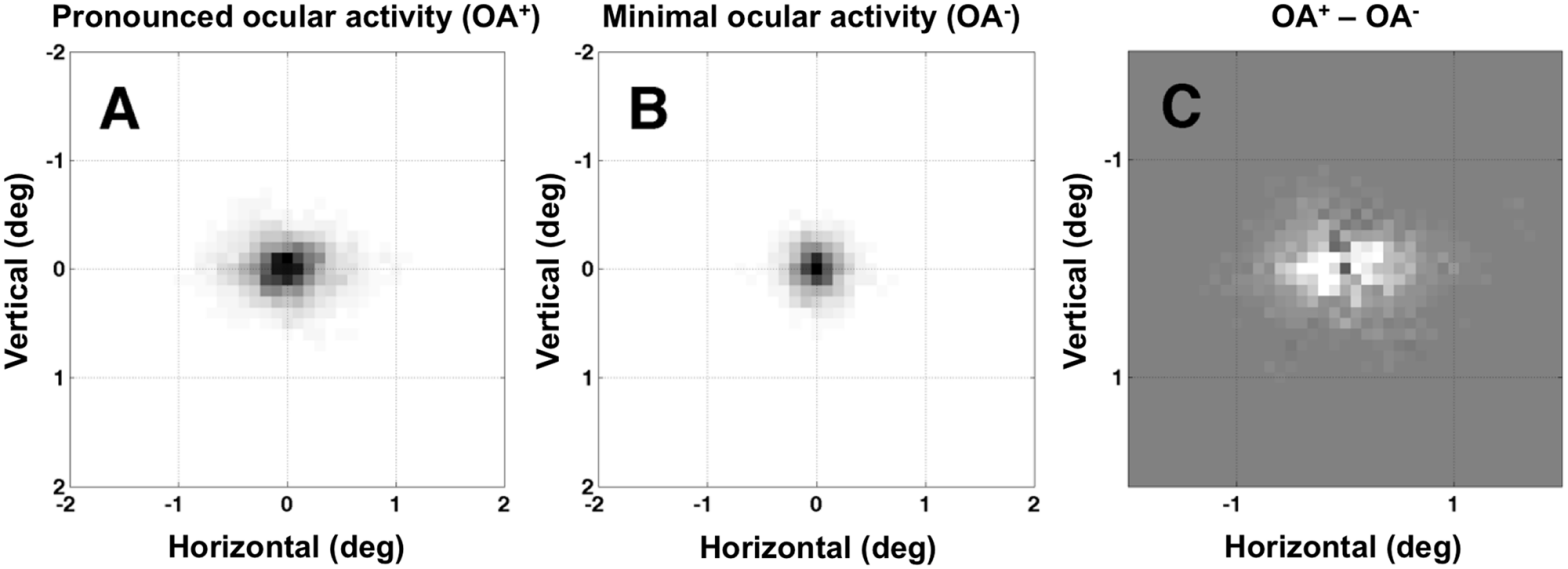
Mean measured gaze direction during trials, averaged across subjects (N = 7). The sampling rate of our equipment (60 Hz) was not sufficient to resolve individual microsaccades, therefore we used a method that separates trials with extreme variance in gaze direction (OA^+^) from those with relatively stable fixation (OA^-^) (see Methods). For each subject, we constructed a two-dimensional matrix with each element representing a 0.1° square region on the LCD display. Horizontal and vertical gaze position measurements were binned into the 0.1° x 0.1° square regions, separately for OA^+^ and OA^-^ trials, and each element of the matrix was assigned the number of times that an eye position was recorded at that location, throughout the entire experiment. Then the grids for all subjects were averaged together to produce panels A and B. (A) Average of trials *with* high variance in measured gaze direction (OA^+^; identified by looking for outliers in the distribution of velocity measurements across trials. (B) Average of all of the remaining trials (OA^-^; i.e. trials *without* high variance in gaze direction). (C) Difference, *with* (OA^+^) minus *without* (OA^-^): lighter colors correspond to locations visited more often on OA^+^ trials, and vice versa for darker colors. Note the dark square in the center, indicating that this location was dominated by OA^-^ trials, and the lighter shades in the periphery indicating that the periphery was dominated by OA^+^ trials. The position data, even *with* high variance in gaze direction, are confined to a radius of < 1°, consistent with the ocular activity in question being fixational eye movements.

Analyses of ocular activity were performed on the 1.5-second time window immediately following stimulus onset, but before a response was made (stimulus duration was 1.33 sec and subjects rarely responded earlier than 300 ms after stimulus cutoff). Pre-processing of the eye-tracking data was carried out using the ILAB software application [30], and further analyses were carried out using MatLab (The MathWorks, Inc). The data were first detrended (linear) to remove slow drift; epochs surrounding large eye movements or eye blinks were replaced with null values (thereby excluding these epochs from any analyses); and then the eye-position data were converted to velocity. The sampling rate of our eye-tracking equipment (60 Hz) was not sufficient to resolve individual microsaccades. Instead we used a method that separates trials with extreme fluctuations in gaze-direction from trials with relatively stable gaze-direction, during the period of time when the subject was instructed to maintain steady fixation. Trials with extreme fluctuations in gaze direction were defined as outliers in the right tail of the distribution of velocity measurements across an entire run ($x\;-\;\tilde{x}\;>\;2\;\times \;iqr$, where $\tilde{x}$ is the median and *iqr* is the inter-quartile range). Trials with one or more such outliers were marked as ocular-activity trials [since we could not rule out minute changes in pupil diameter as a cause of the measured changes in gaze direction, we use the label “ocular activity” rather than “eye movement”]. The variable, OCULAR-ACTIVITY, was thus binary, coded with a 1 (significant variability in gaze direction detected; **OA+**) or 0 (no significant variability in gaze direction detected; **OA-**). The magnitude of within-trial changes in measured gaze direction (< 1) was consistent with these being fixational eye movements (Fig 2).

### Correcting for multiple comparisons

For the post-hoc sliding window analysis (see below) it was necessary to correct for multiple comparisons and temporal non-independence. This was done using a cluster-based permutation test. The analysis was repeated 1000 times shuffling the OCULAR-ACTIVITY trial labels for each subject on each iteration. A sample-wise threshold of p < 0.05 was used to identify positions of the sliding window at which the relevant dependent measure (the difference between ***ϕ***_***AW***_ and ***ϕ***_***CR***_) was significantly different from zero. The largest number of contiguous significant values was recorded on each iteration, and the distribution of these temporal cluster sizes was compared against the temporal cluster sizes in the observed data in order to arrive at a corrected p value.

## Results

**Fig 3:**
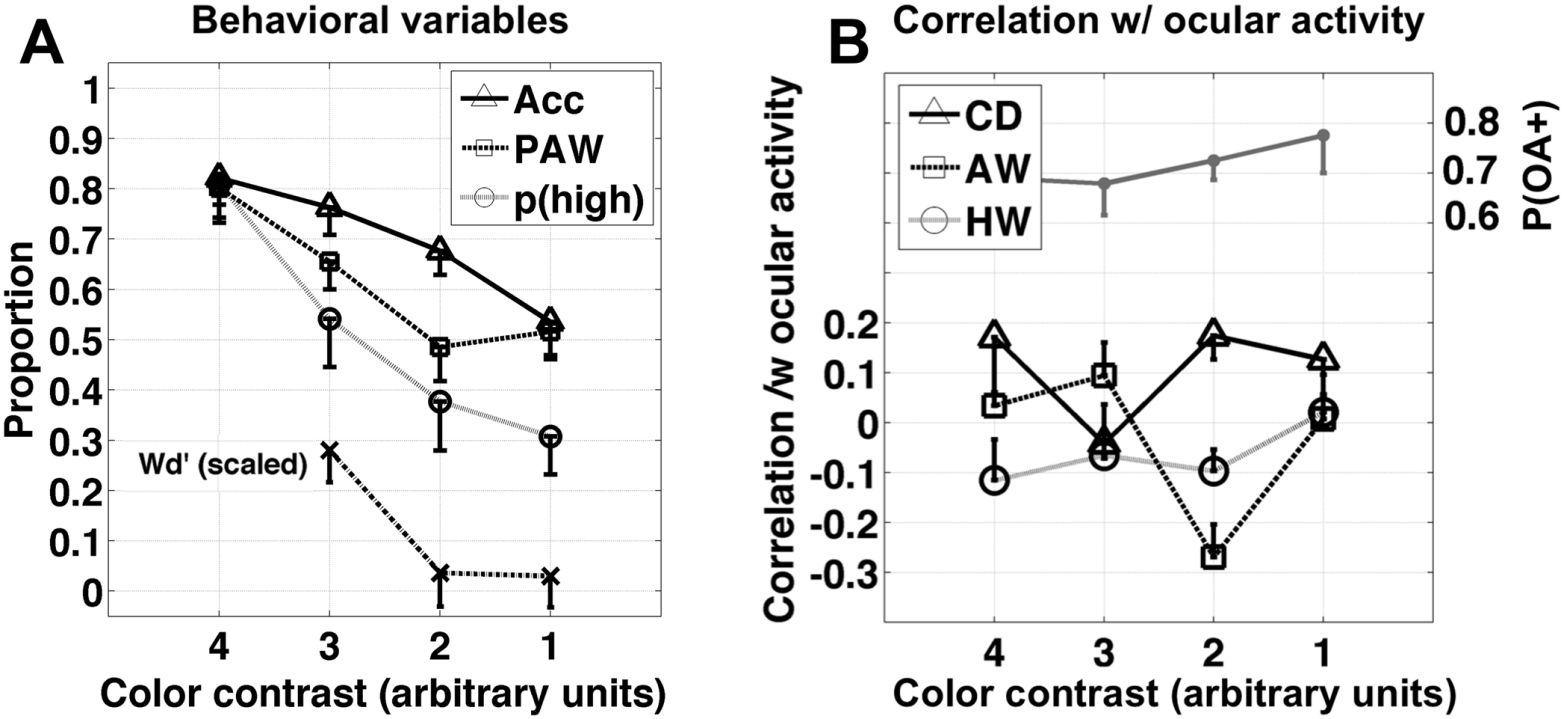
Behavioral and eye-tracking results. (A) proportion correct (solid line w/ triangles), proportion of advantageous wagers (*PAW*, dashed line w/ squares), proportion of high wagers (dotted line w/ circles), and wagering d-prime (***Wd’***, dash-dot line w/ x’s). All measures are proportions, except for ***Wd’***, which is scaled into the range [0,1] for clarity (see Methods). Each subject completed 24 trials at each contrast level. (B) Gray line at the top of panel B shows the mean proportion of OA^+^ trials across subjects (scale on the right). Also shown are the mean correlation between OCULAR-ACTIVITY and CORRECT-RESPONSE (solid line with triangles), the mean correlation between OCULAR-ACTIVITY and ADVANTAGEOUS-WAGER (dashed line with squares), and the mean correlation between OCULAR-ACTIVITY and HIGH-WAGER (dotted line with circles). The mean correlation between OCULAR-ACTIVITY and CORRECT-RESPONSE is significantly greater than zero (p = 0.03, two-sided signed-rank test). The mean correlation between OCULAR-ACTIVITY and ADVANTAGEOUS-WAGER is significantly less than zero (p = 0.015, two-sided signed-rank test). The mean correlation between OCULAR-ACTIVITY and HIGH-WAGER was not different from zero (p = 0.44, two-sided signed-rank test). Abbreviations: CD = “correct decision”, AW = “advantageous wager”, HW = “high wager”.

**Fig 4:**
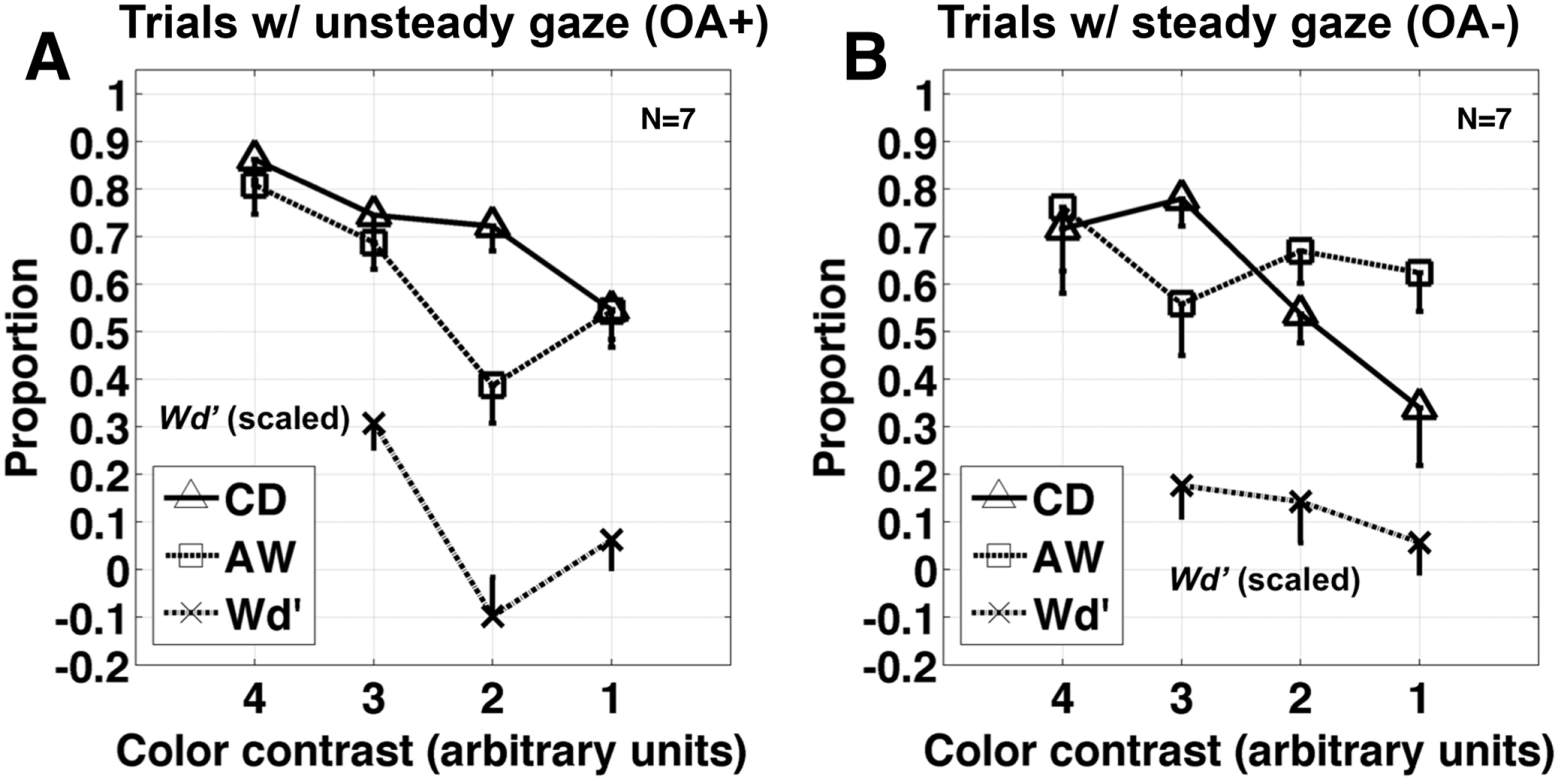
Pattern of results for trials with relatively unsteady (OA^+^) and relatively steady (OA^-^) gaze direction measurements. For each subject, the metrics shown in Fig 3A were computed separately for trials with and trials without detected ocular activity (OA; see Methods). These were then averaged across subjects (N = 7). Labeling of lines is the same as in Fig 3A. OA^+^ trials accounted for approximately 70% of trials on average across subjects (Fig 3B).

The main results are presented in Figs 3 and 4. Looking first at the 7 subjects for whom we had eye-tracker data (Fig 3A): At contrast level 2, accuracy was significantly greater than chance (68% +/- 4.8% SEM, p < 0.02, one-sided Wilcoxon; accuracy > = 50% for 7 of 7 subjects) while neither ***Wd’*** nor meta-***d’*** was significantly different from zero (***Wd’*** = 0.14 +/- 0.23 SEM, ***Wd’*** < 0 for 5 of 7 subjects; Fig 3A). Out of 7 subjects, 3 individually scored significantly higher than chance (i.e. ≥ 17 correct out of 24; p < 0.05 binomial), while ***Wd’*** for each of these 3 subjects, at this same contrast level, was less than zero.

The results were similar if we also included the subjects who contributed only behavioral data (N = 11; S2 Fig). At contrast level 2, accuracy was significantly greater than chance (67% +/- 3.5% SEM, p < 0.005, one-sided Wilcoxon; accuracy > 50% for 9 of 11 subjects) while neither ***Wd’*** nor meta-***d’*** was significantly different from zero (***Wd’*** = 0.18 +/- 0.16 SEM, ***Wd’*** < 0 for 6 of 11 subjects; Fig 3A). In addition, out of 11 subjects, 5 individually scored significantly higher than chance (i.e. ≥ 17 correct out of 24; p < 0.05 binomial). The mean ***Wd’*** for these 5 subjects, at this same contrast level, was not significantly different from zero (0.07 +/- 0.20 SEM; ***Wd’*** < 0 for 3 of the 5). Both ***Wd’*** and meta-***d’*** gave qualitatively similar results when applied to our data—both were not-different-from zero at contrast levels 1 and 2 and both were significantly greater than zero at contrast levels 3 and 4 (S3 Fig).

For the same eleven subjects, reaction times increased monotonically with decreasing color contrast (mean RTs = 440 +/- 50ms SEM, 500 +/- 79ms SEM, 582 +/- 71ms SEM, and 608 +/- 71ms SEM, respectively; N = 11), and the differences were significant according to a one-way repeated measures ANOVA (p = 0.002, F = 6.21, df = 3). There was also a significant slowing of reaction times for incorrect versus correct responses at contrast level 3, where subjects scored on average ~ 75% correct (RT_correct_ = 452 +/- 66ms SEM; RT_incorrect_ = 643 +/- 114ms SEM; p < 0.005 paired-samples signed rank test), but no significant differences in RT at any other contrast level. Notably, there were no significant differences in RT (for correct vs incorrect responses) at contrast level 2, where we found effects tied to eye-tracker measurements.

The authors (AS and MSK) noted from direct observation that small eye movements can disrupt the effectiveness of dichoptic color masking (making contours more visible), even when fusion is not noticeably disrupted. However, disappearance of the object becomes increasingly immune to eye movements at lower levels of color contrast. Thus we conjectured that fixational eye movements (“ocular activity” or OA) might be associated with an increase in accuracy at a color contrast level that is near the subjective threshold of visibility. Accordingly, one would expect that any effect of fixational eye movements would appear at a near-threshold level of color contrast, but not be apparent at lower or higher levels of color contrast due to floor and ceiling effects, respectively.

The trial-by-trial correlation between OCULAR-ACTIVITY (a binary variable, coded with a 1 or 0; see Methods) and correct-response (also a binary variable) was computed as the phi (Φ) coefficient (***ϕ***_***CR***_), which is equivalent to Pearson’s correlation coefficient (***r***) applied to binary data and is interpreted in the same way (Fig 3B). We also computed the correlation between OCULAR-ACTIVITY and high-wager (***ϕ***_***HW***_), and between OCULAR-ACTIVITY and advantageous-wager (***ϕ***_***AW***_), all binary variables. Wagering ***d’*** (***Wd’***) and meta-***d’*** are aggregate measures computed on a set of trials, and so these variables could not be correlated with OCULAR-ACTIVITY on a trial-by-trial basis. As previously noted, the probability that a given wager is labeled as advantageous is partly determined by the subject’s willingness to place high wagers (the wagering criterion). However, the lack of any relationship between OCULAR-ACTIVITY and high-wager argues against an explanation based solely on a shift in the wagering criterion (see below). The results of these analyses are presented in Fig 3B.

Planned tests targeted at contrast level 2 (the contrast level of the dissociation between accuracy and wagering) revealed that OCULAR-ACTIVITY in the 1.5-sec interval following stimulus onset was positively correlated with correct-response (p = 0.03, two-sided signed-rank test; p < 0.05, permutation test; Fig 3B triangles) and negatively correlated with advantageous-wager (p = 0.015, two-sided signed-rank test; p < 0.01, permutation test; Fig 3B squares). There was no significant correlation between OCULAR-ACTIVITY and HIGH-WAGER (-0.098, p < 0.1 signed rank test; Fig 3B circles), suggesting that the effect on advantageous wagering relates to sensitivity rather than a change in criterion. Thus, at a near-threshold level of color contrast, ocular activity was correlated with an increase in the probability of a correct guess and a decrease in the probability of an advantageous wager.

Another way to approach these data is by grouping them into trials with extreme fluctuations in gaze direction (OA^+^) and trials without (OA^-^), and then applying the original analyses separately for each subset. Looking at these results, it is readily apparent that OA^+^ trials account for the gap between object recognition and wagering performance at contrast level 2 (Fig 4). Given that OA^+^ trials accounted for roughly 70% of trials on average across subjects, it is reasonable to conclude that the decoupling of first- and second-order decision making is not simply an effect of dichoptic color masking, but rather is an interaction between dichoptic color masking and fixational eye movements.

**Fig 5:**
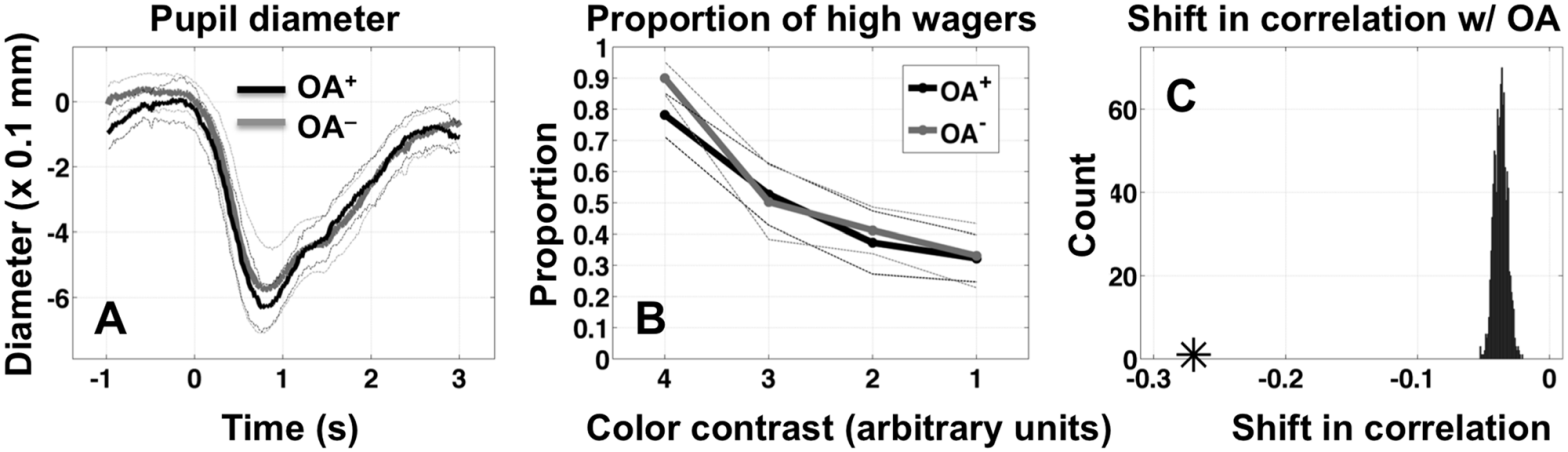
Control analyses. (A) Time course of mean pupil diameter (sampled at 60 Hz) for trials with (OA^+^) and trials without (OA^-^) pronounced ocular activity. There were no significant differences in measured pupil diameter at any point in time from -1.0 to +3.0 seconds relative to stimulus onset. (B) Mean proportion of high wagers across subjects for OA^+^ and OA^-^ trials. There was no difference in the proportion of high wagers at any contrast level, this ruling out criterion artifacts as an explanation for the effect. (C) Expected shift in the correlation between OA^+^ and ADVANTAGEOUS WAGER (AW) driven solely by a an increased correlation between OA^+^ and HIGH WAGER (HW) (narrow distribution to the right, near zero). Black star shows the observed result of -0.27, which is too extreme to be accounted for in this way.

With infrared eye-tracking equipment, small changes in pupil diameter can potentially cause fluctuations in measured gaze direction, and thus what appear to be fixational eye movements may in fact be tied to changes in pupil diameter. We therefore analyzed the pupil diameter data for OA^+^ and OA^-^ trials separately and found no difference in pupil diameter at any time throughout the trial epoch (Fig 5A), consistent with an effect of fixational eye movements. We also re-ran the entire analysis using pupil diameter as the primary covariate rather than gaze direction and this analysis failed to turn up any significant effects. Nevertheless, to err on the side of caution, we make no specific claim as to whether the effect is tied to small eye movements, changes in pupil diameter, or a combination of both. We also found no significant difference in the proportion of high wagers at contrast level 2 for **OA** ^**+**^ and **OA** ^**-**^ trials (0.373 +/- 0.1 SEM, and 0.412 +/- 0.08 SEM, respectively; p = 0.44, two-sided signed-rank test; Fig 5B), reinforcing our claim that the effect is tied to a change in type-2 sensitivity rather than an overall shift in confidence.

Because advantageous-wager is determined jointly by correct-response and high-wager, it is possible that an increasing correlation between OCULAR-ACTIVITY and correct-response (***ϕ***_***CR***_) might necessarily imply a decreasing correlation between OCULAR-ACTIVITY and advantageous-wager (***ϕ***_***AW***_)—i.e. ocular activity may only have an effect on accuracy, with an apparent effect on advantageous wagering as an artifact of the metric. How could an effect of ocular activity on the probability of a correct response influence the correlation between ocular activity and advantageous wagering? If the probability of a correct response increases, but the probability of a high wager stays the same, then the increased number of correct responses will not be met with a proportional increase in high wagers. Thus there will consistently be a few additional correct responses paired with low wagers (considered *not* advantageous), drawing the correlation coefficient downward.

To test whether or not this explanation could account for the negative correlation between eye-movement and advantageous-wager at contrast level 2, we performed a simulation (see S1 Appendix for the computer code) in which we introduced a positive correlation between OCULAR-ACTIVITY and correct-response (***ϕ***_***CR***_) and between OCULAR-ACTIVITY and HIGH-WAGER (***ϕ***_***HW***_) equal to those observed in our data, and then checked the resulting correlation between eye-movement and advantageous-wager (***ϕ***_***AW***_). We recomputed ***ϕ***_***AW***_ 1000 times, with 100 trials in each surrogate experiment, keeping the same proportion of high wagers and **OA** ^**+**^ trials as that observed in the data. On each iteration we computed ***ϕ***_***AW***_ twice, once with ***ϕ***_***CR***_ ≈ 0.2 and ***ϕ***_***HW***_ ≈ -0.1 (the approximate observed values; Fig 3B), and once with both correlations ≈ 0. We used this to build a distribution of the expected shift in ***ϕ***_***AW***_ driven solely by the conjunction [***ϕ***_***CR***_ ≈ 0.2 AND ***ϕ***_***HW***_ ≈ -0.1]. There was indeed a reliable shift in ***ϕ***_***AW***_ as expected (Fig 5C), but the shift (-0.037 +/- 0.005 STD) was very small compared to the observed value of -0.27 (star on the horizontal in Fig 5C) and could not account for the observed effect (p < 0.001, permutation test).

It is also conceivable that a small change in wagering behavior might drive a larger change in the correlation between OCULAR-ACTIVITY and advantageous wagering (***ϕ***_***AW***_; note that the correlation with correct-response would not be affected). To test whether or not this explanation could account for the negative correlation between OCULAR-ACTIVITY and advantageous-wager at contrast level 2, we took the actual data from each of the 7 eye-tracker subjects, shuffled each subject’s face/house responses, *keeping the same total number of high and low wagers*, and then computed the phi correlation between OCULAR-ACTIVITY and advantageous-wager (***ϕ***_***AW***_) as before (see Fig 3B). We repeated this procedure for 1000 random shufflings in order to produce a distribution of ***ϕ***_***AW***_ under the null hypothesis of “chance-level accuracy, observed level of high-wagers”. The percentage of values in the distribution that were more extreme than or equal to the obtained result (-0.27) gives an estimate of the probability of the obtained result under the null hypothesis. One value in the resampling distribution (n = 1000) was more extreme than the obtained phi correlation, so the obtained value is significant at p < 0.002.

### Post-hoc analysis

**Fig 6:**
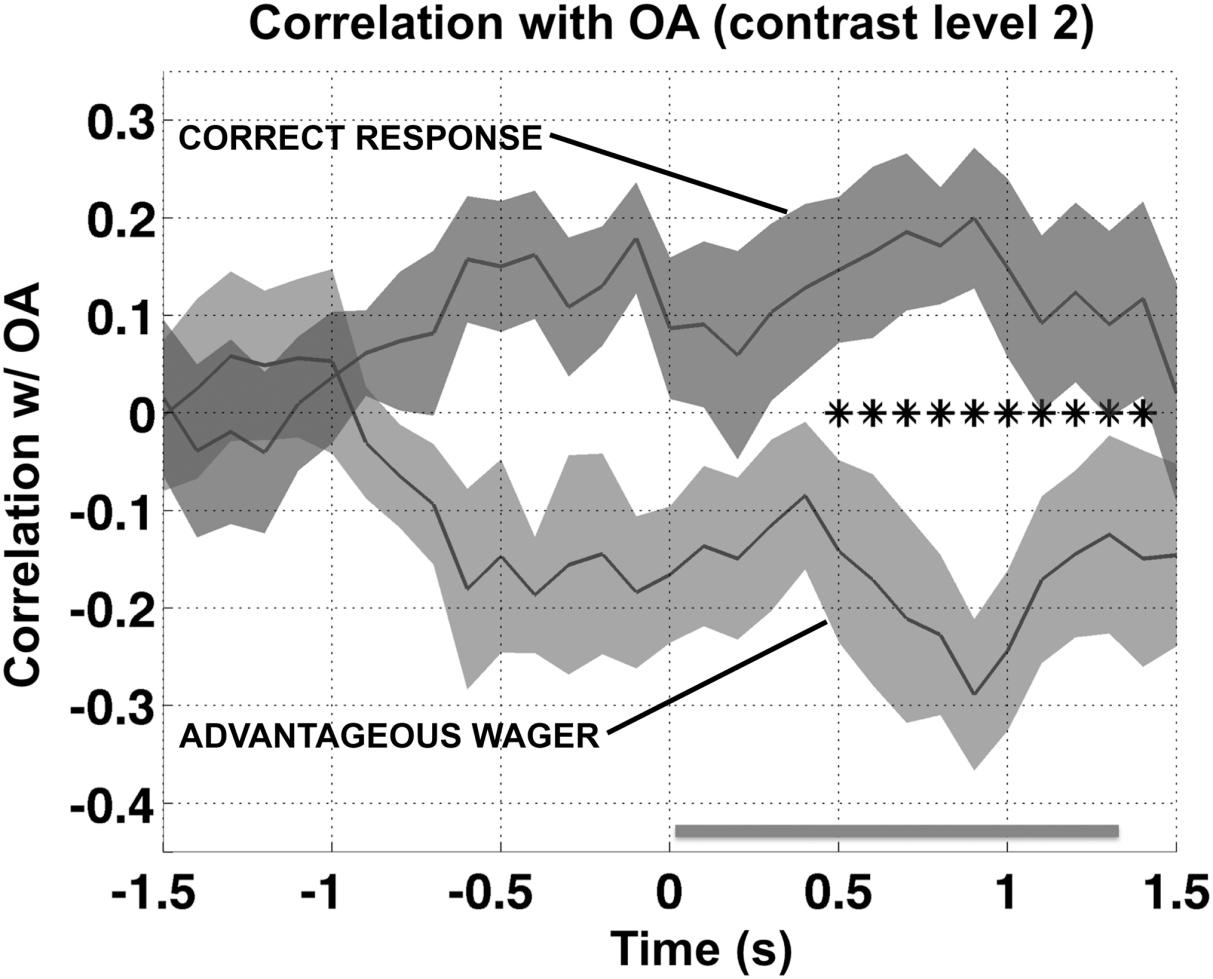
Time course of the effect at contrast level 2. In Fig 3B the correlations between CORRECT RESPONSE and OCULAR ACTIVITY (***ϕ***_***CR***_) and between ADVATAGEOUS WAGER and OCULAR ACTIVITY (***ϕ***_***AW***_) were computed separately at each contrast level using a fixed 1.5-sec time window starting at stimulus onset (0). In the figure above we mapped the time course of the effect at contrast level 2 using a 1.0-second time window centered on the times shown on the horizontal axis. Stars on the horizontal axis show a series of window positions for which the difference between ***ϕ***_***CR***_ and ***ϕ***_***AW***_ was significant (p < 0.01 corrected, signed rank test, N = 7), and the horizontal bar at the bottom shows the time of visual stimulation (from 0 to 1.333 sec). The effect was largest for windows centered at from 0.5 to 1.0 seconds post-stimulus-onset, consistent with an interaction between small eye movements and stimulus processing. The apparent difference before stimulus onset was not statistically significant.

We chose a-priori to test for an effect of ocular activity during the period of time when the stimulus was on the screen. However, the time course of the effect with respect to stimulus and response times can be informative as to the nature of the interaction, and this is not revealed using a fixed time window. Therefore we also performed a systematic post-hoc analysis of the data at different points in time before and during the time of stimulus onset by repeating the analysis in a sliding window. Recall that in the planned analyses ocular activity was computed based on velocity measurements within the 1.5-second time window following stimulus onset (Fig 3B). For the post-hoc analysis we chose to use a 1-second window in order to strike a balance between temporal resolution and sensitivity to the experimental effect (results with a 0.5-second window were noisier, but qualitatively similar). We simply repeated the analysis presented in Fig 3B within a 1-second sliding window centered at every time point from -1.5 to +1.5 seconds, spaced at 100-ms intervals. The resulting time course, computed for color contrast level 2, is presented in Fig 6. We tested for a difference between ***ϕ***_***CR***_ and ***ϕ***_***AW***_ at each time sample using a two-sided signed-rank test and then used a cluster-size permutation test to control for multiple comparisons (p < 0.05 sample-wise threshold). At contrast level 2 the difference between ***ϕ***_***CR***_ and ***ϕ***_***AW***_ was significant, and their signs opposite (***ϕ***_***CR***_ > 0 and ***ϕ***_***AW***_ < 0), for windows centered at 0.5 to 1.4 seconds post-stimulus onset (p < 0.01 corrected; black stars in Fig 6), consistent with an interaction between ocular activity and processing of the visual stimulus (visual stimulation lasted from 0 to 1.333 sec; gray bar at bottom of Fig 6). Thus fixational eye movements and/or changes in pupil diameter that occurred while the stimulus was on the screen were associated with increased type-1 sensitivity and decreased type-2 sensitivity.

## Discussion

With video eye-tracking equipment, because the outline of the pupil is used to infer the direction of gaze, fluctuations in gaze-direction may be confounded with small changes in pupil diameter. However, we carefully analyzed the pupil diameter measurements in our data and found no evidence of any systematic difference in pupil diameter that might explain the effect, and we found no significant effects when we ran the analysis using pupil diameter as the primary covariate. Therefore the results are consistent with an effect of very small stray eye movements made while fixating. It is now well established that fixational eye movements [31], which include microsaccades as well as drift and tremor, play a role in visual perception by enhancing high spatial frequencies [32] and by counteracting neural adaptation in the visual system [33–36]. Without them, steady fixation would cause a static visual scene to fade from view [37, 38].

Our results suggest that type-1 and type-2 decision processes may become decoupled at near-threshold levels of color contrast, where the difference between discrimination accuracy (ACC) and the proportion of advantageous wagers (PAW) is greatest, and that this decoupling is revealed by ocular activity (fixational eye movements, a change in pupil diameter, or a combination of the two) during the time that the stimulus is being presented. According to signal detection theory (SDT) type-2 sensitivity is expected to vary linearly purely as a function of type-1 sensitivity [15], and because type-1 sensitivity (reflected in task performance) may be higher on trials with more ocular activity, this presents itself as a possible confound. However, the default correlation between type-1 and type-2 sensitivity is predicted to be positive, whereas the effect that we report here is of the two diverging in opposite directions. Therefore the observed effect does not yield to a trivial explanation purely in terms of changes in type-1 performance, but rather suggests that the two types of decision making are dissociable and may be subserved by distinct mechanisms.

One might reasonably ask why the effect manifests at a particular level of stimulus contrast and not others. One possible explanation, alluded to earlier, is that, due to floor and ceiling effects, small eye movements may disrupt dichoptic fusion only when the contrast is near threshold: If the contrast is too low, then no amount of perturbation will cause the contours to become visible, and if the contrast is a too high, then the contours may be visible even if gaze is highly stable. Thus, floor and ceiling effects may mask any such effect at anything other than a near-threshold level of color contrast where contours are mostly invisible when gaze is stable, but become slightly visible when gaze is unstable.

In light of prior evidence it would not be at all surprising to find that fixational eye movements [32, 34] and/or changes in pupil diameter [39] might influence task performance. The novel finding here is that, in the context of dichoptic color masking and near-threshold visibility, ocular activity appears to have an opposite relationship with first-order (type-1) and second-order (type-2) visual information processing. Several questions are raised: Is the effect specific to dichoptic color masking, or does it generalize to other manipulations such as low luminance contrast, pattern masking, or low color contrast with binocular viewing? Does the effect depend on the presence of both luminance and chromatic information in the image (we matched luminance using the minimization of subjective flicker, so a slight difference in luminance between figure and ground may have remained)?

It is worth noting, in this context, that recent evidence shows an enhancement of visual sensitivity during smooth-pursuit eye movements for chromatic-, but not for luminance-defined stimuli [40]. In addition, evidence from saccadic suppression suggests that eye movements selectively suppress the magnocellular (M) pathway, leaving the parvocellular (P) processing relatively spared, or even enhanced during saccades [41, 42], and saccadic suppression has also been shown to occur during microsaccades [43, 44]. These observations suggest the intriguing possibility that first-order and second-order visual-perceptual decisions may rely on the M and P pathways to differing degrees, as may be the case for other visual-perceptual faculties. For example, recent research suggests a specific impairment of the M pathway in autism [45–49] and dyslexia [50–52]. In particular, Gori, Cecchini (52] found that among dyslexics, there was a selective impairment of the M pathway in those with poor phonological decoding, but not those with poor lexical decoding, suggesting a division of function. Thus monitoring eye movements while presenting threshold-level stimuli might also find relevance in autism and dyslexia research.

In summary, we have identified a novel property of near-threshold sensory perception, involving an interaction between ocular activity and visually-informed decision making. Although we cannot fully rule out small changes in pupil diameter as a factor, we point out that the main claim of the paper—evidence for a dissociation between type-1 and type-2 decision making—is supported regardless of whether the effect is tied to fluctuations in pupil diameter or fixational eye movements, or both. Further studies are needed to tease apart the relative contribution of fixational eye movements and changes in pupil diameter and determine how either or both of these might interact with sensory processing leading to their observed relationships with accuracy and confidence judgements. Such an approach could be highly informative vis-à-vis the boundary between conscious and non-conscious sensory information processing, and could be very useful as a tool in the study of perception and uncertainty monitoring.

## Supporting Information

S1 Appendix (PDF)

S1 Data This file contains the original behavioral and eye tracker data files. (ZIP)

S1 Fig (PDF)

S2 Fig (PDF)

S3 Fig (PDF)

S1 Table (PDF)
