# Supplementary material for: Paradoxical Interaction between Ocular Activity, Perception, and Decision Confidence at the Threshold of Vision
Source: PLoS One. 2015 May 8;10(5):e0125278. doi: 10.1371/journal.pone.0125278 (PMC4425469; doi:10.1371/journal.pone.0125278)
Supplement: S1 Appendix — (PDF) [file pone.0125278.s001.pdf]

Paradoxical interaction between ocular activity, perception, and meta-cognition at the threshold of vision

Schurger A, Kim M, & Cohen JD

S5 Appendix

Computer code for simulation

The simulation was performed using MatLab (version 7.7.0.471, R2008b, The MathWorks, Inc.). Lines that begin with a '%' sign are comments and are shown in green (the style of the MatLab text editor). The code below (on the following pages) can be pasted into a file called simulate.m and then called from the MatLab command line.

```
function results = simulate(nExperiments)
%
% function results = simulate(nExperiments)
%
% This simulates what happens if you introduce a correlation between
% accuracy and eye movements. Does this make the correlation between
% advantageous wagering and eye movements go more negative? If so, then by
% how much? I will use H0 to stand for "without correlation between
% accuracy and eye movements", and H1 will stand for "with correlation
% between accuracy and eye movements.
%
% The simulation is run many times in order to produce a distribution, by
% calling the sub-function run_one_experiment.
%
% The result is, yes there does seem to be a tendency for the correlation
% between advantageous wagers and eye movements to go a bit negative as the
% correlation between eye movements and correct responses goes up. But the
% effect is very small and cannot reasonably account for the effect
% in our data.
%
if nargin==0
    nExperiments = 1000;
end

results = zeros(nExperiments,1);
for i=1:nExperiments
    if mod(i,10)==0
        disp(sprintf('Experiment %03d...',i))
    end
    [rB0,rB1] = run_one_experiment;
    results(i) = mean(rB1)-mean(rB0);
end

figure
hist(results,max(floor(nExperiments./20),5));
ax=axis; axis([-0.35 0.1 ax(3) ax(4)]);
set(gca,'fontsize',18,'fontweight','bold')

return

end

%%%%%%%%%%%%%%%%%%%%%%%%%%%%%%%%%%%%%%%%%%%%%%%%%%%%%%%%%%%%%%%%%%%%%%%%%%%%

function [rB0,rB1] = run_one_experiment



nTrials = 100;
emFactor_a = [0, 0.2]; % influence of eye movement on accuracy [H0, H1]
emFactor_b = [0, -0.3]; % influence of eye movement on high bet [H0, H1]

rA0 = zeros(nTrials,1); % correlation (r) between eye movement and accuracy under H0
rA1 = zeros(nTrials,1); % correlation (r) between eye movement and accuracy under H1
rB0 = zeros(nTrials,1); % correlation (r) between eye movement and advantageous
betting under H0
rB1 = zeros(nTrials,1); % correlation (r) between eye movement and advantageous
betting under H1

for i=1:100
    eyeMvmt = double(rand(100,1)>0.30); % ~70% eye movement trials
    hibet_src = double(rand(100,1)>0.6); % source for simulated wagers (~40% high)
    acc_src = rand(100,1); % source for simulated accuracy
```

```
63
64          % eyeMvmt is either zero or one, so it determines whether or not
65          % emFactor is applied
66          acc = double((acc_src + emFactor_a(1)*eyeMvmt)>0.5);
67          hibet = double((hibet_src + emFactor_b(1)*eyeMvmt)>0.5);
68          adbet = double(acc==hibet);
69          r = corrcoef(acc,eyeMvmt);
70          rA0(i) = r(1,2);
71          r = corrcoef(adbet,eyeMvmt);
72          rB0(i) = r(1,2);
73
74          % emFactor_a of 0.2 boosts accuracy to ~70% correct, consistent with our
75          % data...
76          acc = double((acc_src + emFactor_a(2)*eyeMvmt)>0.5);
77          hibet = double((hibet_src + emFactor_b(2)*eyeMvmt)>0.5);
78          adbet = double(acc==hibet);
79          r = corrcoef(acc,eyeMvmt);
80          rA1(i) = r(1,2);
81          r = corrcoef(adbet,eyeMvmt);
82          rB1(i) = r(1,2);
83      end
84
85      return
86
87      end
88
89
```
