# Supplementary material for: Paradoxical Interaction between Ocular Activity, Perception, and Decision Confidence at the Threshold of Vision
Source: PLoS One. 2015 May 8;10(5):e0125278. doi: 10.1371/journal.pone.0125278 (PMC4425469; doi:10.1371/journal.pone.0125278)
Supplement: S1 Fig — (PDF) [file pone.0125278.s003.pdf]

Paradoxical interaction between ocular activity, perception, and meta-cognition at the threshold of vision

Schurger A, Kim M, & Cohen JD

S1 Figure

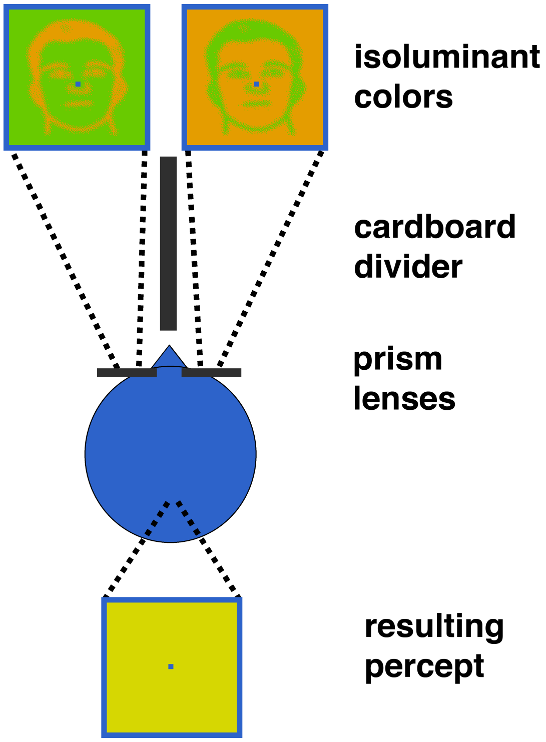

S1 Figure: Stereoscopic stimulus presentation method, illustrating "opposite color" stimuli. (Moutoussis and Zeki 2002, Schurger 2009)

References

Moutoussis, K. and S. Zeki (2002). "The Relationship Between Cortical Activation and Perception Investigated With Invisible Stimuli." Proc Natl Acad Sci USA **99**(14): 9527-9532.

Schurger, A. (2009). "A very inexpensive MRI-compatible method for dichoptic visual stimulation." J Neurosci Methods **177**(1): 199-202.
