# Supplementary material for: Paradoxical Interaction between Ocular Activity, Perception, and Decision Confidence at the Threshold of Vision
Source: PLoS One. 2015 May 8;10(5):e0125278. doi: 10.1371/journal.pone.0125278 (PMC4425469; doi:10.1371/journal.pone.0125278)
Supplement: S2 Fig — (PDF) [file pone.0125278.s004.pdf]

Paradoxical interaction between ocular activity, perception, and meta-cognition at the threshold of vision

Schurger A, Kim M, & Cohen JD

S3 Figure

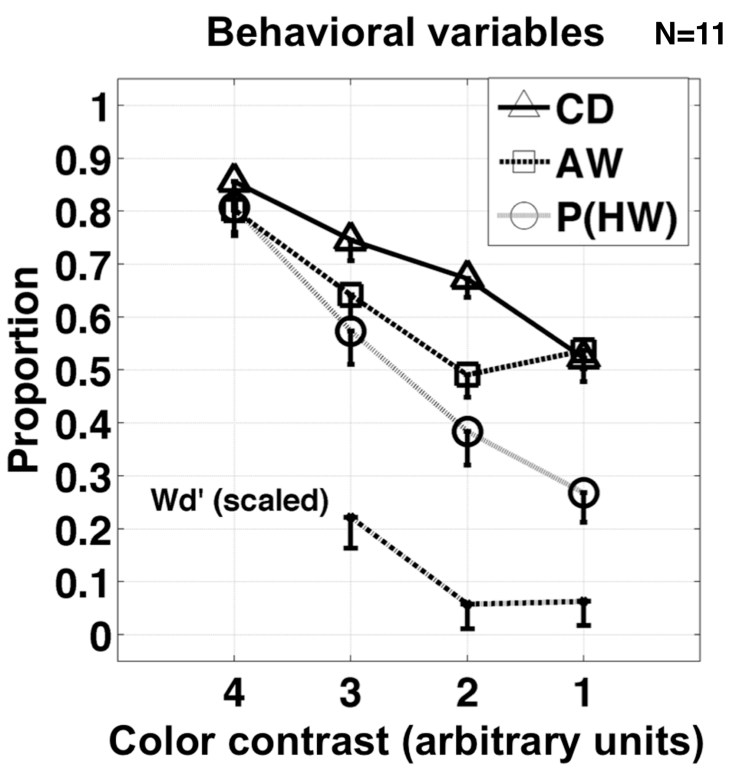

Figure S2: Behavioral results incuding four additional subjects who contributed only behavioral data.

Proportion correct (solid line w/ triangles), proportion of advantageous wagers (***PAW***, dashed line w/ squares), proportion of high wagers (dotted line w/ circles), and wagering d-prime (***Wd'***, dash-dot line w/ x's). All measures are proportions, except for ***Wd'***, which is scaled into the range [0,1] for clarity (see Methods). Each subject completed 24 trials at each contrast level (N=11).
