# Supplementary material for: Paradoxical Interaction between Ocular Activity, Perception, and Decision Confidence at the Threshold of Vision
Source: PLoS One. 2015 May 8;10(5):e0125278. doi: 10.1371/journal.pone.0125278 (PMC4425469; doi:10.1371/journal.pone.0125278)
Supplement: S3 Fig — (PDF) [file pone.0125278.s005.pdf]

Paradoxical interaction between ocular activity, perception, and meta-cognition at the threshold of vision

Schurger A, Kim M, & Cohen JD

S4 Figure

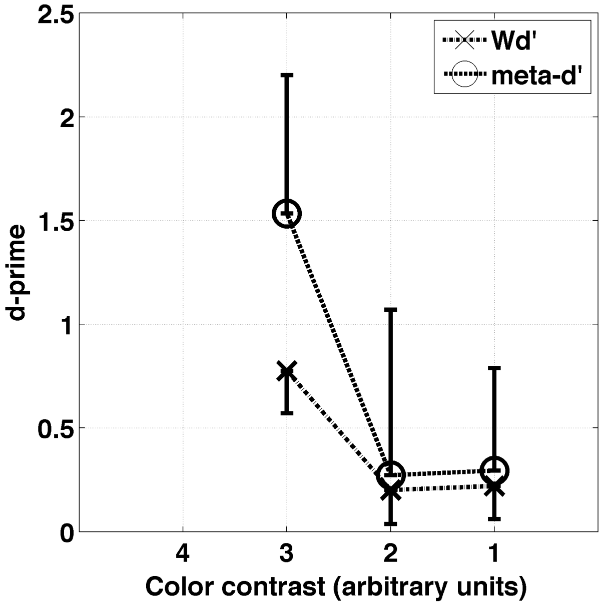

Figure S4: Comparison of wagering ***d'*** (***Wd'***) and meta-***d'***.

Average wagering-***d'*** (dash-dot with x's) and meta-***d'*** (dashed with o's) for the 11 subjects who contributed behavioral data. The two measures give qualitatively similar results: both are significantly greater than zero at contrast level 3, and both are not different from zero at contrast levels 2 and 1 (there were too few incorrect responses and/or low wagers at contrast level 4 to accurately estimate either). Wagering ***d'*** (***Wd'***) is computed by treating each correct response as a "target", and each instance of a correct response combined with a high wager as a "hit". ***Wd'*** is then equal to *zinv*(TPR) – *zinv*(FPR), where *zinv* is the inverse normal distribution, TPR is the true-positive (hit) rate, and FPR is the false-positive (false-alarm) rate. This measure has been referred to as "type-2 ***d'***" in the literature [refs] and is calculated in precisely the same way that ***d'*** is calculated according to signal-detection theory [ref], except that the "targets" are one's own correct responses to sensory stimuli, rather than the sensory stimuli themselves. Type-2 ***d'*** depends in part on the type-1 ***d'***. That is to say, sensitivity to one's own correct responses will appear to increase, according to type-2 ***d'***, as the fraction of correct responses increases, even if "real" sensitivity to one's own correct responses does not change. In order to address this confound [Maniscalco & Lau] developed a measure called "meta-***d'***" that estimates the amount of information that is available to metacognition, in the same units as the traditional ***d'***.
