# Supplementary material for: Paradoxical Interaction between Ocular Activity, Perception, and Decision Confidence at the Threshold of Vision
Source: PLoS One. 2015 May 8;10(5):e0125278. doi: 10.1371/journal.pone.0125278 (PMC4425469; doi:10.1371/journal.pone.0125278)
Supplement: S1 Table — (PDF) [file pone.0125278.s006.pdf]

Paradoxical interaction between ocular activity, perception, and meta-cognition at the threshold of vision

Schurger A, Kim M, & Cohen JD

S2 Table

RGB values for the 7 color pairs used in our experiments. Note that these were calibrated for our display device and are thus unlikely to be isoluminance on a display device other than the one on which they were calibrated. RGB values are in the range 0 <= RGB <= 1. Each shade of green was computed by taking the symmetric RG values of its corresponding orange (e.g. [0.2745 0.4 0] / [0.4 0.2745 0]) and multiplying it by a coefficient β (0 < β <= 1) that yielded minimal subjective flicker when calibrating the display device using heterochromatic flicker photometry (see Methods).

Shades of **green**				Shades of **orange**		
**R**	**G**	**B**		**R**	**G**	**B**
0.2343	0.3414	0		0.4	0.2745	0
0.2636	0.3538	0		0.4	0.2980	0
0.2889	0.3638	0		0.4	0.3176	0
0.2993	0.3678	0		0.4	0.3255	0
0.3204	0.3756	0		0.4	0.3412	0
0.3419	0.3832	0		0.4	0.3569	0
0.3528	0.3869	0		0.4	0.3647	0
